# Controlled van der Waals epitaxy of 2D single-crystal molecular film

**DOI:** 10.1093/nsr/nwae405

**Published:** 2024-11-25

**Authors:** Xinghui Liu, Young Hee Lee

**Affiliations:** Science and Technology on Aerospace Chemical Power Laboratory, Laboratory of Emergency Safety and Rescue Technology, Hubei Institute of Aerospace Chemotechnology, China; Center for Integrated Nanostructure Physics (CINAP), Sungkyunkwan University, South Korea; Department of Physics, Sungkyunkwan University (SKKU), South Korea; Center for Two-Dimensional Quantum Materials, Hubei University of Technology, China

Two-dimensional (2D) materials have garnered significant attention for their unique properties, which enable advances in both fundamental physics and next-generation electronic devices [1]. However, the large-scale production of high-quality 2D films, essential for semiconductor applications, remains a major challenge. Recent developments in van der Waals (vdW) epitaxy have made it possible to grow single-crystal, layer-structured 2D atomic crystals, such as graphene on single-crystal Cu(111) [2] and Cu/Ni(111) substrates [3], and wafer-scale MoS₂ [4], WS₂ [5], and WSe₂ [6] by step-edge induced growth on sapphire. These successes have demonstrated the potential for industrial-scale integration of 2D materials. However, unlike atomic crystals that are held together by strong in-plane covalent bonds, molecular crystals feature weaker vdW forces in vertical dimension, making it difficult to control ordered 2D structure formation. As a result, the epitaxial growth of high-quality molecular 2D crystals, particularly on large scales, remains a significant challenge. This study explores these complexities and emerging strategies aimed at overcoming the limitations in the large-area growth of molecule-based 2D materials.

**Figure 1.**
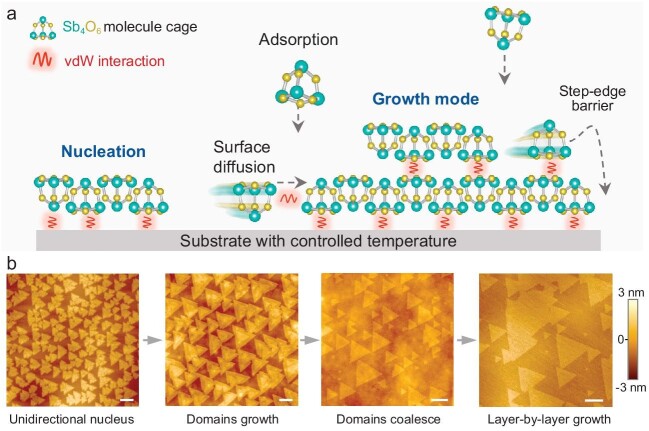
(a) Schematic of growth modes and molecule behavior during vdW epitaxial growth of Sb_2_O_3_ film. (b) Time evolution of AFM images with typical stages of growth of single-crystal Sb_2_O_3_ film. Scale bar, 200 nm. Adapted from Ref. [7].

In a recent breakthrough, researchers from Tianyou Zhai's group have developed a novel method to grow single-crystal 2D Sb₂O₃ molecular films using vdW epitaxy, addressing longstanding challenges in the controlled growth of molecular 2D crystals [7]. By carefully controlling the nucleation and growth stages, the study demonstrates how lattice matching between the Sb₂O₃ layer and the substrate plays a crucial role in determining the orientation of crystal nuclei (Fig. 1). The researchers show that substrates such as hexagonal boron nitride (hBN) and graphene can facilitate the formation of highly ordered, orientation-controlled Sb₂O₃ nuclei with minimal formation energy. Interestingly, the low symmetry of the hBN substrate enhances this effect by breaking the energy degeneracy, which allows for the selective growth of unidirectional Sb₂O₃ nuclei under temperature-controlled conditions. The study reports an impressive alignment consistency of >98% across large areas. Additionally, the researchers highlight the importance of overcoming the Schwoebel-Ehrlich (E-S) barrier in enabling layer-by-layer growth, offering thermodynamic and kinetic insights into how the E-S barrier can be overcome to optimize the growth process for vdW epitaxy.

The high-quality Sb₂O₃ films were successfully integrated as dielectric layers in 2D MoS₂-based field-effect transistors (FETs), showcasing their potential for next-generation electronic applications. The exceptional dielectric properties of the Sb₂O₃ films led to a remarkable reduction in leakage current, with a four-order-of-magnitude improvement compared to devices made from polycrystalline Sb₂O₃ films. Furthermore, the high-quality vdW interface between the Sb₂O₃ dielectric and the MoS₂ channel significantly enhanced the device's performance. Notably, the devices exhibited outstanding switching characteristics, including a theoretical subthreshold swing of 60 mV/dec and an on-off current ratio exceeding 10⁷, all within an operating voltage of just 0.5 V. These results highlight the Sb₂O₃ films’ promise in enabling ultra-scaled, low-power electronic devices, particularly in the context of 2D materials.

Overall, this study represents a pioneering advancement in the controlled growth of single-crystal 2D molecular films through vdW epitaxy, achieving unprecedented precision in both thermodynamic and kinetic control. The researchers push beyond traditional epitaxial techniques by carefully aligning the interactions between molecules and the substrate, overcoming the challenges posed by weak intermolecular forces. This breakthrough enables precise control over molecular assembly and crystal quality, which is crucial for the large-scale fabrication of high-quality 2D molecular films. The insights gained from this work not only deepen our understanding of molecular film growth but also open up new possibilities for the use of 2D molecular crystals in high-performance, ultra-scaled electronic devices, paving the way for their integration into future technologies.
